# Biosurveillance: A Review and Update

**DOI:** 10.1155/2012/301408

**Published:** 2012-01-02

**Authors:** Nicholas E. Kman, Daniel J. Bachmann

**Affiliations:** ^1^Ohio Task Force 1-FEMA Urban Search and Rescue, Department of Emergency Medicine, The Ohio State University Medical Center, 4813 Cramblett Hall, 456 West Tenth Avenue, Columbus, OH 43210, USA; ^2^Emergency Medicine, The Ohio State University Medical Center, 4734 Cramblett Hall, 456 West Tenth Avenue, Columbus, OH 43210, USA

## Abstract

Since the terrorist attacks and anthrax release in 2001, almost $32 billion has been allocated to biodefense and biosurveillance in the USA alone. Surveillance in health care refers to the continual systematic collection, analysis, interpretation, and dissemination of data. When attempting to detect agents of bioterrorism, surveillance can occur in several ways. Syndromic surveillance occurs by monitoring clinical manifestations of certain illnesses. Laboratory surveillance occurs by looking for certain markers or laboratory data, and environmental surveillance is the process by which the ambient air or environment is continually sampled for the presence of biological agents. This paper focuses on the ways by which we detect bioterrorism agents and the effectiveness of these systems.

## 1. Introduction

Since the terrorist attacks of September 11, 2001, and the anthrax release in the following month, there has been a heightened interest in bioterrorism surveillance. The years immediately following these attacks were met with increased awareness and funding from the federal government. This paper will focus on the methods that we can use to prepare ourselves and detect these bioagent attacks.

The anthrax attacks of 2001, the SARS outbreak in 2004, and the recent H1N1 Influenza outbreak remind us that an essential component of preparedness for bioterrorism includes surveillance methods that can detect and monitor the course of an outbreak and thus minimize associated morbidity and mortality [1]. Surveillance of a population can be achieved in several ways. Syndromic surveillance occurs by monitoring clinical manifestations of certain illnesses. This type of surveillance occurs when health-related data, like* International Classification of Diseases Ninth Revision* (ICD-9) codes, are analyzed to signal possibility of an outbreak. Laboratory surveillance occurs by looking for certain markers or laboratory data. The Laboratory Response Network (LRN) is the United States' laboratory system for detecting, confirming, and reporting bioterrorism agents. Within the LRN, sentinel laboratories are tasked with singling out suspicious specimens for further testing in higher-tier labs. Environmental surveillance is the process by which the ambient air or the environment is continually sampled for the presence of biological agents [2].

Unfortunately, the practice of bioterrorism surveillance remains poorly studied. A recent systematic review of 29 biosurveillance systems concluded that there is insufficient evidence to determine which of these systems is best [1]. One thing is known. Whether it is an astute clinician like the one who made the first diagnosis of anthrax in 2001 or the complex chemical lab techniques that are used to detect plague, we must maintain our ability to identify and respond to a biologic terrorist attack.

## 2. Background

Surveillance is recognized as the single most important public health instrument for identifying public health events of global concern, particularly infectious diseases that are emerging [3]. Not only is the use of surveillance helpful for bioterror attacks, but also the information generated by surveillance systems is also useful in the recognition and response to emerging infectious diseases. These epidemics are not related to traditional bioterror agents but their public health significance can be equally alarming. The recent H1N1 Influenza outbreak is a prime example of this.

 The four functions of basic surveillance include (1) detecting cases of disease in specific populations and reporting the information, (2) analyzing and confirming reported case information to detect outbreaks, (3) providing timely and appropriate responses at the local/regional level to allow appropriate national level prevention and control of disease outbreaks, and (4) providing epidemiologic intelligence information to assist in long-term management of public health and health-care policies and programs [3].

 Surveillance in health care refers to the continual systematic collection, analysis, interpretation, and dissemination of data [4]. Early methods of public health surveillance have been passive and voluntary. This process occurred when patients were diagnosed with a reportable communicable disease and local health departments were notified by clinicians, hospitals, or laboratories. Time would pass as information meandered through local and state health departments. Although many of the key components of surveillance occur at the local level, it takes many working parts for this to occur in a timely fashion.

Passive surveillance is an important component to global biosurveillance. It has the advantages of being inexpensive, easy to implement, and free of technologic barriers. However, it likely is not rapid and accurate enough to be used alone to respond to a bioterrorist attack. Passive surveillance is used best with other methods to quickly identify the treat and institute public health protection measures such as immunization, prophylaxis, and quarantine.

Active surveillance is the method of tracking emerging infectious disease threats. Active surveillance involves outreach to actively collect disease information from specific groups, such as sentinel medical providers or hospitals. Typically, active surveillance is undertaken to look for a specific disease. Active surveillance is more labor intensive and requires more public health resources than passive surveillance [5].

Systems for bioterrorism surveillance for public health require 3 key features: timeliness, high sensitivity and specificity, and routine analysis of data [1]. Timeliness of diagnosis is vital as the effectiveness of most treatments hinges on early detection. To this end, the electronic collection and reporting of surveillance data has improved detection as compared with manual methods [1]. High sensitivity is necessary as, without this, systems may fail to detect cases of bioterrorism-related illness which could result in delays in detection. On the opposite end, systems with inadequate specificity may have frequent false alarms, which will result in costly public health responses. Using the example of a food-borne illness outbreak, a system with low sensitivity may miss the sentinel cases and not identify the trend until the outbreak is already widespread. This compromises the ability of the surveillance system to adequately mount an effective public health response to the outbreak. Using the same example, a system with low specificity may identify cases which are not truly related to an outbreak resulting in an unnecessary public health response with diversion of resources from other true outbreaks. Sensitivity and specificity are typically inversely related such that optimization of one characteristic is at some expense to the other. Striking the optimal balance between these two characteristics for any given surveillance system is difficult [1].

## 3. Syndromic Surveillance

The first key to identifying a potential bioterrorism event is to maintain a strong index of suspicion. The initial cases of West Nile Fever Virus in 1999 and the deliberate release of anthrax in 2001 were ultimately diagnosed by astute clinicians working hand-in-hand with lab technicians, not by public health surveillance systems. Syndromic data are gathered before laboratory results are reported; therefore, health departments may be able to recognize increases in disease incidence before formal diagnoses are made and to respond to outbreaks early in their course. For this reason, the CDC, state and local public health agencies, and the US Government and military have invested heavily in syndromic surveillance.

Methods of syndromic surveillance include many clues and data points which public health personnel can use to identify patterns. Data sources such as nurse hotline calls, over-the-counter medication purchases, and chief complaints from emergency-department visits can monitor illness clusters [6]. Some other clues to suspicious events include sharp rises in the frequency or severity of communicable diseases, including those in animals. Additional red flags include an unusual cluster or age distribution, occurrence of rare diseases, presence or lack of exposure history, travel to an endemic location, unexplained deaths, or pathogens with unusual antimicrobial resistance [7].

In response to the events of 2001, new types of surveillance systems were developed to detect epidemics through population-based reporting of symptoms tracked by time and region [8]. Many cities and states in the United States use syndromic surveillance, which monitors nonspecific, prediagnostic indicators for disease outbreaks in near real-time to provide an early warning of infectious disease outbreaks in their communities. Syndromic surveillance systems (SSS) monitor descriptive data from clinical diagnoses, chief complaints, and behaviors (e.g., school and work absenteeism, illness-related 911 calls, emergency room admissions for symptoms indicative of infectious disease) to infer patterns suggestive of an outbreak [9]. A comparison of syndromic surveillance with traditional clinical recognition is presented in Table 1 [10].

The most important determinants of detection for any given SSS were analyzed in a methodological review of 35 evaluations of outbreak detection in automated SSSs [11]. These determinants are key to taking one or more high-volume data feeds and differentiating the outbreak cases or “signal” from the baseline cases or “noise.” The determinants were subdivided into characteristics of the system and characteristics of the outbreak being monitored. While evaluations using natural outbreaks were best suited to answer qualitative questions, simulated outbreaks were also useful to allow greater flexibility and increased quantitative results [11].

The influential system characteristics identified included representativeness or sampling approach of the system, the outbreak detection algorithm, and the specificity of the algorithm. For example, systems that monitor a larger proportion of the population have a higher sensitivity for detecting an outbreak. Similarly, systems that only monitor one type of clinical setting—such as ED visits only—were less sensitive. Furthermore, the studies that relied on simulated outbreaks suggested that temporal surveillance was more sensitive when the algorithm considered multiple days of data at each decision point versus data from each day individually. Important determinants related to the outbreak included magnitude and shape of the signal and timing of the outbreak. Intuitively, signals with a rapid rise over a short period of time improved outbreak detection as compared with those that rose more slowly over time. The ideal magnitude of the signal for consistent detection is not clear. The studies indicated magnitudes ranging from 10% up to as much as 60%. Similarly, the influence of the timing of the signal was not consistent, though there was a better detection when the outbreak occurred in context of a lower baseline of activity [11]. Based on these characteristics, one could envision an ideal SSS that monitored a large population at multiple clinical venues over multiple days at a time and flagged signals with rapid rise over a low baseline to at least a magnitude of 10%.

Almost immediately after the terrorist attacks of September 11, 2001, The New York City Department of Health and Mental Hygiene (NYCDOHMH) collaborated with the CDC to initiate an emergency-department-based syndromic surveillance for agents [12]. The system looked for symptoms that could be associated with a bioagent release such as respiratory distress, rash, gastrointestinal symptoms, neurologic impairment, and sepsis. Providers filled out forms with patient data that were analyzed by epidemiologists. This system was up and running in 15 New York City ED's within 2 days of its conception.

Syndromic surveillance systems monitor health care utilization patterns using data collected in real time, usually electronically. One example of a SSS is the Electronic Surveillance System for the Early Notification of Community-Based Epidemics (ESSENCE), which automatically downloads ICD-9 codes from U.S. Department of Defense health care facilities [1]. This novel use of ICD-9 codes is one way to group patient visits into syndromes. There are more than 10,000 ICD-9 codes available [13]. Patient visits are grouped by ESSENCE algorithms into one of eight syndromes based on lists of selected ICD-9 codes. If an increase in number of visits for a syndrome is noted, the clinic can be contacted for more information and an investigation can be launched.

Started in November of 2003, BioSense is a CDC Internet-based syndromic surveillance application designed for the early detection of intentional and natural infectious disease outbreaks [12]. BioSense receives data electronically from several sources. The Department of Veterans Affairs and Department of Defense provide ICD-9 codes for visits to their facilities. Retail pharmacies provide sales information on over-the-counter medications, and Laboratory Corporation of America provides information on laboratory tests ordered. After examination by CDC analysts, public health officials can access their summary reports.

Current SSSs monitor the average pattern of patients reporting to primary care physicians or emergency-departments and signal an alarm whenever the pattern changes. Reporting sources include emergency-departments, intensive care units, hospital admission and discharge systems, and laboratories [8]. The Rapid Syndrome Validation Project (RSVP) relies on physicians to enter data on patients presenting with a syndrome of interest into a computer that has a touch-screen interface with RSVP [14].

The Emergency Department is the most common clinical source for surveillance data, though other sources of data have been proven to be useful. The Real-time Outbreak and Disease Surveillance Laboratory (RODS) Pennsylvania is the biosurveillance system for the Commonwealth of Pennsylvania. In production since 1999, it monitors 3 million visits to emergency rooms from 137 emergency-departments a year and simultaneously monitors 1262 retail stores in Pennsylvania for disease outbreaks. By utilizing the National Retail Data Monitor (NRDM), they have found a strong correlation that exists between the purchase of over-the-counter (OTC) medications and emergency room visits for constitutional illnesses. This information is useful for predicting coming epidemics as the tracking patterns of influenza and seasonal gastrointestinal illnesses often precede trends in hospital data [15]. One study demonstrated that OTC electrolyte sales preceded hospital visits for gastrointestinal and respiratory illnesses by 2.4 weeks [16]. 

The Connecticut Department of Public Health has been effectively using an SSS based on unscheduled hospital admissions since 2001. The Hospital Admission Syndromic Surveillance (HASS) system monitors 32 Connecticut-based acute-care hospitals with required reporting for eleven syndromic categories. Daily monitoring of data with weekly comprehensive analysis allows identification of disease clusters and routine public health followup for further action or response [17].

Syndromic surveillance efforts have been expanded to include outpatient monitoring also. This type of system takes advantage of the experience of ambulatory care physicians, who are also likely to be among the first to encounter patients during the prodrome of any potential bioterrorism-related illness. One such system developed with a private large ambulatory multispecialty group practice in Eastern Massachusetts demonstrated that surveillance coverage of 5–10% of a region's population may be adequate to detect significant clusters of interest. Several ideal components of this particular system included the automated collection of information, the use of preexisting data from a standard healthcare database, and the minimal cost for its implementation and continuous administration [18].

Although most systems for syndromic surveillance are continuously collecting, analyzing, and reporting data, some systems are designed for short-term use at mass-gatherings thought to be terrorist targets. These SSSs are referred to as event-based or “drop-in” surveillance [1]. One such “drop-in” surveillance system studied by the Bioterrorism Preparedness and Response Program demonstrated fair-to-good agreement of patient classification into an appropriate syndrome category when comparing use of Emergency Department chief complaints to discharge diagnoses. The findings were suggestive that use of discharge diagnoses may increase surveillance validity for “drop-in” and even possibly automated surveillance systems [19]. It is thought that syndromic surveillance systems are best used synergistically with laboratory surveillance.

## 4. Alternative Surveillance Systems

The Centers for Disease Control and Prevention (CDC) has pioneered surveillance systems for monitoring other indicators of disease beside the traditional symptom- and diagnosis-based data used for clinical and syndromic surveillance. One such system is the Early Aberration Reporting System (EARS). This is a free tool which has been utilized and modified in both cities (Boston, NYC, Los Angeles) and in state public health agencies (Georgia, Florida, Tennessee, North Carolina, and Mississippi). It uses nontraditional public health data sources including school absenteeism rates, over-the-counter medication sales, 911 calls, veterinary data, and ambulance run data [20]. 

One novel epidemiologic surveillance approach has been developed by Google Inc and the CDC during the influenza season of 2007-08. This system monitored the health-seeking behavior of millions of users per day in the form of queries to online search engines. Ginsberg et al. demonstrated use of their model to estimate influenza-like illness within 85–96% of CDC-reported actual illness prevalence for the mid-Atlantic region of the USA. The advantages of this internet-based system were that illness statistics were available with a reporting lag of only one day, compared to the 7–14 day lag of CDC surveillance reports [21].

Though the Google surveillance system was specifically designed to monitor for influenza-like illness, the concept is more broadly applicable to other infectious pathogens such as bioterror agents. In addition to earlier detection of outbreaks, other advantages include freely available information to both the public and the government officials, automated processing with near real-time dissemination, and relative inexpensiveness for operation. Unfortunately, the specificity of internet-based surveillance remains unclear and could create more issues related to a high false-positive rate. These systems also require large populations with adequate internet access across regions and socioeconomic classes [22].

Another emerging example of a web-based surveillance system is the HealthMap Project. This collaborative undertaking performs extraction, categorization, filtration, and integration of aggregated reports from multistream real-time internet surveillance data [23]. The round-the-clock process involves automated data mining assisted by analyst review and reclassification. This system specifically focuses on identifying the “breaking news” trends to avoid overwhelming public health officials with low-impact problems [23]. The HealthMap system was applied to the H1N1 outbreak of 2009 with impressive results. The time difference between report of suspected cases and confirmed cases of H1N1 influenza was tracked by country with an overall median lag time of 12 days [24]. This time period can and will significantly alter the impact of the subsequent public health response. Further integration of these types of innovative systems with more traditional surveillance offers the greatest promise for future surveillance of emerging diseases [24].

The true utility of the SSSs is the dissemination and integration of its main output: surveillance data. In 2007, the CDC's Office of Critical Information Integration and Exchange created of the CDC created the BioPHusion Center with a mission to provide a CDC-wide resource that facilitates the exchange, integration, and visualization of relevant information from a variety of sources to enhance agency and programmatic situational awareness for decision-making and early event detection. Its goal is to share timely and actionable information to public health programs and leaders at the national, state, local, tribal, and global levels. They use data from a wide variety of governmental, private, and other sources to create an integrated daily report of potential events available through their Public Health Information Integration Portal [25]. Other publicly accessed CDC resources for information exchange include the Epidemic Information Exchange (Epi-X) and the Public Health Information Network (PHIN).

## 5. Laboratory Surveillance

Clinical laboratories have been the cornerstone of diagnosis in infectious diseases of public health importance. In 1999, the CDC, the Federal Bureau of Investigation (FBI), and the Association of Public Health Laboratories (APHL) established the Laboratory Response Network (LRN) of about 120 laboratories [26]. The RODS laboratory is an example of one such system used for active surveillance as well as research efforts in the field of biosurveillance [15]. The mission of the LRN is to maintain an integrated network of laboratories that are fully equipped to respond to acts of chemical or biological terrorism, emerging infectious diseases, and other public health emergencies [26, 27]. In addition to identifying agents, the LRN is responsible for developing protocols for the handling, identifying, and reporting of potential biological agents to other national security agencies [2].

The LRN includes federal laboratories (CDC), state and local public health labs, military labs (the United States Army Medical Research Institute for Infectious Diseases (USAMRIID)), food testing (FDA), environmental laboratories, veterinary laboratories (United States Department of Agriculture), and international laboratories (Canada, the United Kingdom, and Australia) [2]. The laboratories involved in the LRN are divided into levels A through D, based on capabilities and function. Table 2 describes the levels of labs and their function [2, 26, 27].

As described in Table 2 above, sentinel laboratories are the first tier of the LRN and are responsible for sorting through their daily routine clinical tests to find suspicious biothreat specimens. The response to a local outbreak is the first and, perhaps, most important level. Sentinel laboratories must operate using Biosafety Level 2 procedures and possess a class II certified biological safety cabinet [27]. These labs are staffed by workers with only basic sentinel lab training. When a suspected biothreat agent is identified by one of these workers, it is sent to the local and state public health labs that comprise the second tier of the LRN. These reference laboratories then perform rapid confirmatory testing while maintaining biosafety level (BSL-3) facilities [7]. Once this threat is confirmed, it is passed on to the third tier in the LRN, the national laboratories. The national laboratories are equipped with the most secure containment labs (BSL-4) that they can use if necessitated by the agent.

Effective communication between the laboratory divisions is essential to preparedness. Ongoing dialogue between clinicians, sentinel laboratories, and LRN reference laboratories is essential to confirm the diagnosis quickly. In the USA, if a sentinel laboratory cannot rule out a bioterrorism agent, then it must be referred to an LRN reference laboratory [7]. For cases garnering high suspicion, state public officials are typically contacted and specimens are transported under the jurisdiction of law enforcement.

The following principles described by Pien et al. guide clinicians with respect to sentinel laboratory evaluation of potential bioterrorism agents [7]. (1) The initial evaluating physician should obtain optimum specimen collection instructions from the sentinel laboratory and alert them to the possibility of dangerous pathogen. (2) To maximize speed, accuracy, and safety, sentinel laboratories should limit culture manipulation to what is required by LRN reference labs. (3) Labs are to not inoculate highly suspected smallpox, hemorrhagic fever viruses, alphaviruses, or any unknown viral agents of potential bioterrorism into cell culture. Local public health authorities or the CDC should be contacted prior to collection. (4) Labs are directed to not send environmental (e.g., packages, powders, letters, soil, or water), food, animal, or plant specimens to sentinel laboratories for analysis. Instead, these should be referred directly to a LRN reference laboratory. (5) Finally, to reduce the risk of laboratory-acquired infection, restrict manipulation of certain potential agents (e.g., *Francisella tularensis, Brucella *species, *Coxiella burnetii, Burkholderia mallei, *and *Burkholderia pseudomallei*) to environments under certified class II biological safety cabinet or BSL-3 conditions [7].

The CDC is responsible for monitoring the reference labs via regular proficiency training [30]. It cannot do the entire job alone and delegates some of the work to states local municipalities. This introduces some pitfalls and inconsistencies to the LRN. Many states and locals have different laws regulating this reporting. Additionally, as some of the sentinel labs are privately owned, only moderate oversight can occur at this level. To this end, a recent survey showed that only 73.8% of reporting labs indicated that they had sufficient personnel, equipment, and training to respond to a bioterrorism event [27]. Another study performed exercises with three category A organisms of bioterrorism (Anthrax, Plague, and Tularemia). In this study, sentinel laboratories only correctly identified 84% of bioterrorism agents [26]. This study showed that sentinel lab performance is improving, but still not likely at this optimal goal.

## 6. Environmental Surveillance

There are two categories of environmental detection systems currently in existence, the remote or standoff detection of aerosol clouds and the point detection systems of the environment [2].

### 6.1. Remote Detection Systems

One way that remote detection systems monitor for potential biothreats from a distance is by the observation of aerosolized masses or clouds. Finding and evaluating the contents of a cloud is referred to as “standoff” detection [28]. On its most basic level, these detectors aim to alert military or civilian public health personnel to the presence of an approaching cloud. After the initial identification of the cloud, a more detailed assessment of the contents, such as water droplets, inert inorganic material, dead biotic particulates, or nonpathogenic microbes, is pursued [28]. Remote or standoff detection surveillance systems include cloud recognition by Doppler radio and radar, the Army's long- and short-range biological standoff detection systems. The Army's standoff detection systems are capable of detecting aerosol clouds from long distances, as well as determining their composition using ultraviolet light reflectance [2].

### 6.2. Point Detection Systems

Point detection systems are those that sample an environmental source, attempting to detect and identify the agent. Specific identification of a biologic agent by rapid diagnostics at the site of the attack can be done using immunologic assays, genetic assays, and mass spectrometry [2]. These systems can further be differentiated by the type and location of sample collected. For example, The Interim Biological Agent Detector is used on US naval ships to monitor the air for an increase in particulate concentrations [1]. Biowatch is an example of an environmental detection system that takes aerosol samples from locations in fixed sites, such as airports or public buildings.

### 6.3. Biowatch

In July of 2003, the Department of Homeland Security (DHS), the Environmental Protection Agency (EPA), and the CDC introduced the Biowatch program—a federal monitoring system intended to speed detection of specific biological agents that could be released in aerosolized form during a biological attack. Biowatch air sampling devices are deployed in 31 major U.S. cities. The air samples typically are tested daily for signs of the particular biological agents being monitored [2, 29].

The core purpose and intent of Biowatch is to hasten the public health response to a covert bioattack. This would allow rapid distribution of medical countermeasures, like antibiotics or vaccinations, thereby saving lives [30]. To this end, there are 500 air filters in these 31 urban areas that work as Biowatch sensors. These sensors have also been deployed to select indoor venues and are used to monitor mass-gathering events, such as the Super Bowl. This nationwide surveillance system uses distributed aerosol collectors to capture airborne ppapers onto removable dry filters that are transported daily to LRN laboratories for analysis [30]. An expanded deployment of the same technology in 2005 was referred to as Generation 2 Biowatch. Generation 2 Biowatch reportedly can sample and report detection from 10 to 36 hours [31]. Biowatch sensors are intended to be integrated into a complex network of environmental monitoring, medical surveillance activities, and public health response. It is thought that this integration of public awareness information, as well as syndromic, laboratory, and environmental surveillance technologies and systems, would be the best defense against a bioterrorist attack [2, 31].

Biowatch is not perfect. As currently operated, Biowatch filters are collected every 24 hours and delivered to local laboratories, where they are analyzed according to prescribed protocols. If this analysis recognizes one of the five biothreat agents that the system is designed to detect, it is termed a Biowatch Actionable Result (BAR). Laboratories report BARs to local public health officials, who must then decide how to respond. This decision is not taken lightly. The decision to treat a BAR as evidence of a bioattack could have huge consequences if it were a false alarm, including destructive impacts on the community's confidence in the public health system. Since 2003, there have been a number of BARs, though none have been the result of a biological attack. In some BAR cases, Biowatch samples contained material that was genetically similar to that found in Biowatch target organisms. These cases turned out to be from microbes that are present in the ambient environment but do not represent a threat to humans. Progress has been made in developing lab tests that distinguish these close relatives of bioweapons and work on more specific lab assays is ongoing [31].

 Warnings from Biowatch would only be timelier than current health care systems under specific circumstances. Those are if a large-scale aerosol attack were to use certain biological agents and occur where Biowatch is deployed and if Biowatch successfully detects the biological agent [29].

 Generation 3 Biowatch is currently in development. The next evolution of environmental sensor technology has been referred to as a “lab in a box” [31]. Gen 3 Biowatch would be more sophisticated than the current Biowatch sensors, with the ability to automatically collect outdoor air samples, perform molecular analysis of the samples, and report the results electronically to provide near-real time reporting. The target requirements for Generation 3 are reduction of time to diagnosis to 4 hours, increasing targeted biothreat agents monitored, reducing unit procurement costs down to $80,000 per detector unit, and detection sensitivity and false-positive rates remaining consistent with the current system's performance [31].

## 7. Cost/Benefit

A bioterrorist incident is considered a low probability but high-cost event. The costs are high, because many agents go undetected until the onset of symptoms when treatment is less effective and more expensive [32]. That said, it is not economically feasible to the government to undertake a blanket deployment of biosensors [2]. Despite the growing cost, Congress continues to pass legislation intended to strengthen the nation's biological surveillance by increasing funding of federal and state biological surveillance. A 2007 DHS report documented that since the events of 2001, almost $32 billion has been allocated to biodefense and biosurveillance in the USA alone [3].

As of 2005, Biowatch costs per year were approximately $13,672,096. This figure includes labor costs, site upgrades, supplies, travel, training, and other operation and maintenance costs [32]. Most agree that this cost is justified if the probability of a bioterrorism incident remains high as the benefits of Biowatch improve.

As the Biowatch network is presently planned to expand with greater capability, this will increase the costs of the Biowatch Generation 3 system as compared to the currently deployed Generations 1 and 2 systems. Considering the operational complexity of current US biosurveillance systems, it is imperative that the operational advantages and feasibility of the proposed system be carefully evaluated and that actual performance of Generation 3 be tested in field conditions before large technology acquisition investments are made. The Department of Homeland Security will continue to work collaboratively to conduct and oversee developmental and operational tests of Biowatch 3 [31].

## 8. Limitations/Current Challenges

Despite the massive increase in funding and resources that has catapulted our capability for increased biosurveillance over the past decade, both in the US and abroad, there remain several challenges that must be addressed. The value of disease surveillance systems to public health officials is greatest when several systems are used together. The primary limitation of disease surveillance at this point is the limited coordination and lack of interoperability among the various private and federal surveillance systems.

As part of the 9/11 Commission Act, the National Biosurveillance Integration Center (NBIC) was created within the Department of Homeland Security to integrate information and support an interagency biosurveillance community. A 2009 report from the US Government Accountability Office on the state of biosurveillance and resource use concluded that there exists confusion, uncertainty, and skepticism around the value of the interagency community, as well as the mission and purpose of the NBIC within that community. Furthermore, there was a lack of clarity about roles, responsibilities, joint strategies, policies, and procedures for operating across agency borders [33].

Each individual system provides useful information, though no single system is complete [5]. Since it is possible to travel to most places in the world in less time than the incubation period for many infectious diseases, our networks must be expanded to allow for global surveillance [9]. The World Health Organization produced a major overhaul of their International Health Regulations in 2005 with a specific focus on the coordination of the global public health response to natural disasters, accidental release, or deliberate use of biological and chemical agents that can affect global public health [3]. But this cooperation must exist at every level—local, state, federal, and international—to maximize the effects of surveillance.

Another challenge of our current surveillance approach is the consequences of false-positive activation. These systems must be designed for high sensitivity given the overlap of commonplace pathogens with potential bioterror agents. Unfortunately, this may often sacrifice the specificity of the systems. The false alarms may be due to technical malfunctions or to naturally occurring events, such as the detection of anthrax in areas with large concentrations of cattle [32]. The subsequent mobilization of significant resources is not only costly but can be very distracting and generate overwhelming public distress.

 The acquisition of data to fuel a surveillance system, especially the syndromic and clinical-based ones, may be challenged by concerns for privacy of protected health information (PHI). Though the Health Insurance Portability and Accountability Act (HIPAA) Privacy Rule allows for essential exchanges of health data during a public health emergency, the flow of PHI may be slowed by misunderstandings of the Privacy Rule's accounting requirement. This obstacle regarding HIPAA exceptions requires education of the necessary parties prior to the event of a bioterrorist attack [34].

## 9. Conclusions

Halting the spread of a bioterrorism attack will take a combination of the surveillance systems described above. It is only through active study, proper funding, and creative invention that we will be able to improve these systems.

## Figures and Tables

**Table 1:** Characteristics of bioterrorism-related epidemics that affect detection through clinical recognition versus syndromic surveillance.

Characteristics^a^	Clinical recognition^b^	Syndromic surveillance^c^
Duration and variability of incubation period	Broader distribution of incubation period increases likelihood that patients with short incubation-period disease would be diagnosed before a statistical threshold of syndromic cases is exceeded.	More narrow distribution of incubation period which leads to a steeper epidemic curve in the initial phases increase likelihood that statistical threshold would be exceeded sooner.
Duration of nonspecific prodromal phase	Shorter prodrome increases likelihood of recognition or diagnosis at more severe or fulminant stage.	Longer prodrome increases likelihood that increase in syndromic manifestations would be detectable and that recognition of more severe stage (at which a diagnosis is more apt to be made) would be delayed.
Presence or absence of clinical sign that would heighten suspicion of diagnosis	Presence increases likelihood of earlier clinical recognition and diagnosis (e.g., mediastinal widening on chest X-ray in inhalational anthrax or multiple cases of rare disease presenting at similar time).	Absence decreases likelihood that diagnosis would be considered clinically, increasing opportunity for earlier detection by means of syndromic surveillance.
Likelihood of making diagnosis in the course of routine clinical evaluation	If diagnosis is apt to be made in the course of a routine diagnostic evaluation (not dependent on clinical suspicion of specific bioterrorism infection), early diagnosis through clinical care is likely.	If diagnosis is dependent on the use of a special test that is unlikely to be ordered in the absence of clinical suspicion of diagnosis, then diagnosis in clinical care may be delayed, increasing the opportunity for early detection through syndromic surveillance.

^a^Infection or disease attributes that may affect detection of an epidemic.

^b^Increases likelihood of initial detection through routine clinical care and reporting.

^c^Increases likelihood of initial detection through syndromic surveillance.

**Table 2:** Laboratory divisions within the laboratory response network.

Level A Laboratory (Sentinel Labs)	Tier 1	Approximately 2300 hospital and clinic labs were likely first to receive specimens. Role is to rule out and refer to a lab within LRN to confirm a diagnosis.
Level B Laboratory (Reference Labs)	Tier 2	Increased capabilities to confirm diagnoses of biological agent. County public health labs where role is confirmatory testing, initial susceptibility testing, and referral.
Level C Laboratory (Reference Labs)	Tier 2	Much like level B, State public health labs that confirm diagnosis and refer to national laboratory. There are approximately 160 reference labs (B and C).
Level D Laboratory (National Labs)	Tier 3	National laboratories whose primary responsibility is to further characterize the agent (CDC, USAMRIID have biosafety level IV (BSL-4) capabilities).

## References

[1] Bravata DM, McDonald KM, Smith WM (2004). Systematic review: surveillance systems for early detection of bioterrorism-related diseases. *Annals of Internal Medicine*.

[2] Karwa M, Currie B, Kvetan V (2005). Bioterrorism: preparing for the impossible or the improbable. *Critical Care Medicine*.

[3] Castillo-Salgado C (2010). Trends and directions of global public health surveillance. *Epidemiologic Reviews*.

[4] Persell DJ, Robinson CH (2008). Detection and early identification in bioterrorism events. *Family and Community Health*.

[5] Sell TK (2010). Understanding infectious disease surveillance: Its uses, sources, and limitations. *Biosecurity and Bioterrorism*.

[6] Uscher-Pines L, Farrell CL, Babin SM (2009). Framework for the development of response protocols for public health syndromic surveillance systems: case studies of 8 US states. *Disaster Medicine and Public Health Preparedness*.

[7] Pien BC, Saah JR, Miller SE, Woods CW (2006). Use of sentinel laboratories by clinicians to evaluate potential bioterrorism and emerging infections. *Clinical Infectious Diseases*.

[8] Sintchenko V, Gallego B (2009). Laboratory-guided detection of disease outbreaks: three generations of surveillance systems. *Archives of Pathology and Laboratory Medicine*.

[9] Choffnes ER (2008). Improving infectious disease surveillance. *Bulletin of Atomic Scientists*.

[10] Buehler JW, Berkelman RL, Hartley DM, Peters CJ (2003). Syndromic surveillance and bioterrorism-related epidemics. *Emerging Infectious Diseases*.

[11] Buckeridge DL (2007). Outbreak detection through automated surveillance: a review of the determinants of detection. *Journal of Biomedical Informatics*.

[12] Borchardt SM, Ritger KA, Dworkin MS (2006). Categorization, prioritization, and surveillance of potential bioterrorism agents. *Infectious Disease Clinics of North America*.

[13] Betancourt JA, Hakre S, Polyak CS, Pavlin JA (2007). Evaluation of ICD-9 codes for syndromic surveillance in the electronic surveillance system for the early notification of community-based epidemics. *Military Medicine*.

[14] Zelicoff A, Brillman J, Forslund DW (2001). *The Rapid Syndrome Validation Project (RSVP)*.

[15] Real-time Outbreak and Disease Surveillance Laboratory at the University of Pittsburgh. https://www.rods.pitt.edu/site/content/blogsection/9/69/.

[16] Hogan WR, Tsui F-C, Ivanov O (2003). Detection of pediatric respiratory and diarrheal outbreaks from sales of over-the-counter electrolyte products. *Journal of the American Medical Informatics Association*.

[17] Dembek ZF, Carley K, Siniscalchi A, Hadler J (2004). Hospital admissions syndromic surveillance—Connecticut, September 2001-November 2003. *Morbidity and Mortality Weekly Report*.

[18] Lazarus R, Kleinman K, Dashevsky I (2002). Use of automated ambulatory-care encounter records for detection of acute illness clusters, including potential bioterrorism events. *Emerging Infectious Diseases*.

[19] Fleischauer AT, Silk BJ, Schumacher M (2004). The validity of chief complaint and discharge diagnosis in emergency department-Based syndromic surveillance. *Academic Emergency Medicine*.

[20] Early Aberration Reporting System (EARS) Centers for Disease Control and Prevention. http://emergency.cdc.gov/surveillance/ears/.

[21] Ginsberg J, Mohebbi MH, Patel RS, Brammer L, Smolinski MS, Brilliant L (2009). Detecting influenza epidemics using search engine query data. *Nature*.

[22] Wilson K, Brownstein JS (2009). Early detection of disease outbreaks using the Internet. *CMAJ*.

[23] Brownstein JS, Freifeld CC, Reis BY, Mandl KD (2008). Surveillance sans frontières: internet-based emerging infectious disease intelligence and the HealthMap project. *PLoS Medicine*.

[24] Brownstein JS, Freifeld CC, Chan EH (2010). Information technology and global surveillance of cases of 2009 H1N1 influenza. *New England Journal of Medicine*.

[25] Rolka H, Walker D, Heitgerd J (June 2009). An Overview of CDC’s OCIIX BioPHusion Program. *Integrated Surveillance Seminar Series*.

[26] Wagar EA, Mitchell MJ, Carroll KC (2010). A review of sentinel laboratory performance identification and notification of bioterrorism agents. *Archives of Pathology and Laboratory Medicine*.

[27] Kalish BT, Gaydos CA, Hsieh YH (2009). National survey of Laboratory Response Network sentinel laboratory preparedness. *Disaster Medicine and Public Health Preparedness*.

[28] Kosal M The basics of chemical and biological weapons detectors: research story of the week. http://cns.miis.edu/stories/031124.htm.

[29] Institute of Medicine Biowatch and Public Health Surveillance: Evaluating Systems for the Early Detection of Biological Threats. http://www.iom.edu/Reports/2010/BioWatch-Public-Health-Surveillance-Evaluating-Systems-Early-Detection-Biological-Threats.aspx.

[30] Regan JF, Makarewicz AJ, Hindson BJ (2008). Environmental monitoring for biological threat agents using the autonomous pathogen detection system with multiplexed polymerase chain reaction. *Analytical Chemistry*.

[31] Department of Homeland Security (DHS) Testimony of Tara O’Toole Before the House Subcommittee on Homeland Security Appropriations, on Biosurveillance. http://www.dhs.gov/ynews/testimony/testimony_1271436311919.shtm.

[32] Schneider H (2005). Protecting public health in the age of bioterrorism surveillance: is the price right?. *Journal of Environmental Health*.

[33] GAO report on biosurveillance Developing a collaboration strategy is essential to fostering interagency data and resource sharing. http://www.gao.gov/new.items/d10171.pdf.

[34] Hodge JG, Brown EF, O'Connell JP (2004). The HIPAA privacy rule and bioterrorism planning, prevention, and response. *Biosecur Bioterror*.

